# Identification of Novel *ARSB* Genes Necessary for p-Benzoquinone Biosynthesis in the Larval Oral Secretion Participating in External Immune Defense in the Red Palm Weevil

**DOI:** 10.3390/ijms21051610

**Published:** 2020-02-26

**Authors:** Yu-Chen Pu, Xin-Yu Liang, He Zhang, Hua-Jian Zhang, Li-Na Xu, Ya-Nan Ji, Shu-Ning Huang, Juan Bai, You-Ming Hou

**Affiliations:** 1 State Key Laboratory of Ecological Pest Control for Fujian and Taiwan Crops, Fujian Agriculture and Forestry University, Fuzhou 350002, Fujian, China; fafupuyuchen@163.com (Y.-C.P.); yuyu9662@163.com (X.-Y.L.); zhhe1205@163.com (H.Z.); huajianzhang@fafu.edu.cn (H.-J.Z.); kunka-lina@outlook.com (L.-N.X.); yananji1@outlook.com (Y.-N.J.); blinghsn@163.com (S.-N.H.); bj8492765@163.com (J.B.); 2 Fujian Provincial Key Laboratory of Insect Ecology, College of Plant Protection, Fujian Agriculture and Forestry University, Fuzhou 350002, Fujian, China

**Keywords:** arylsulfatase B, external immune secretion, gene expression profile, inhibitory efficiency, p-benzoquinone, *Rhynchophorus ferrugineus*, salivary gland

## Abstract

External secretions, composed of a variety of chemical components, are among the most important traits that endow insects with the ability to defend themselves against predators, parasites, or other adversities, especially pathogens. Thus, these exudates play a crucial role in external immunity. Red palm weevil larvae are prolific in this regard, producing large quantities of p-benzoquinone, which is present in their oral secretion. Benzoquinone with antimicrobial activity has been proven to be an active ingredient and key factor for external immunity in a previous study. To obtain a better understanding of the genetic and molecular basis of external immune secretions, we identify genes necessary for p-benzoquinone synthesis. Three novel *ARSB* genes, namely, *RfARSB-0311*, *RfARSB-11581*, and *RfARSB-14322*, are screened, isolated, and molecularly characterized on the basis of transcriptome data. To determine whether these genes are highly and specifically expressed in the secretory gland, we perform tissue/organ-specific expression profile analysis. The functions of these genes are further determined by examining the antimicrobial activity of the secretions and quantification of p-benzoquinone after RNAi. All the results reveal that the *ARSB* gene family can regulate the secretory volume of p-benzoquinone by participating in the biosynthesis of quinones, thus altering the host’s external immune inhibitory efficiency.

## 1. Introduction

Insects are the most diverse group of animals and, remarkably, include more than a million described species, exceeding half of all known living organisms and inhabiting almost every environment on earth [1]. To endow them with the ability to live in a wide range of ecological environments and adjust to various adversities, including attack by natural enemies, an external immune defense via chemical secretions is one of their most critical and important traits [2,3,4]. Moreover, many external chemical secretions have repellent or irritant properties [5]. Several coleopterans, including *Tribolium castaneum* [3,6], *Tribolium confusum* [7], and *Tenebrio molitor* [8], are especially prolific producers of benzoquinone and a variety of substituted benzoquinone compounds to exert immunological functions in vitro.

Some preliminary studies have reported the in vivo biosynthetic pathways in *Eleodes longicollis* of quinones, which are secreted into external environments, and that alkylated benzoquinones are formed by acetate condensation and the p-benzoquinone is generated from pre-formed aromatic rings of amino acids, including tyrosine and phenylalanine [9]. All p-quinones are usually present in exocrine glands in the form of phenolic β-glucosides, which are then transferred to the inner part of the gland and form active quinones via a series of enzymatic reactions [10]. However, no data are available on the genes involved in these processes. Among these quinones, p-benzoquinone is especially unstable, reactive, and highly toxic, requiring highly efficient detoxication systems [6]. Therefore, arthropods, including insects, have also evolved multiple auto-detoxication mechanisms to reduce the toxic effects of these secretions on individuals for immune purposes [4,6]. For example, tenebrionid is protected from its own toxic quinones in the external secretions by cuticular linings both internally and externally [6,11]. The *Tribolium* beetle possesses the ability to rapidly partition the secretions away from the somatic cells, firstly by producing the secretions in the cuticle-lined organelles and then keeping them in storage sacs that are formed from invaginations of the cuticle [6,9,10]. In addition, *E. longicollis* can also degrade partial p-benzoquinone into tyrosine via reductase in vivo [9].

Arylsulfatases are the catalytic enzyme family that can hydrolyze various sulfates including glucosaminoglycan (GAG), sulfatide (SFT), and sulfated steroids [6,12,13,14,15]. These proteins have highly homologous amino acid sequences but exhibit different functions and substrate specificities and distinct action sites in cells [12,14,15,16,17]. A member of this family, known as arylsulfatase B (ARSB), has the ability to hydrolyze large sugar molecules and sulfate macromolecules [6,13,14]. Because of its hydrolytic potential, ARSB can enhance resistance in *Escherichia coli* against heavy metals such as arsenic and antimony [18,19,20]. Furthermore, in *Staphylococcus aureus*, it not only strengthens the resistance against arsenite and antimonite but also acts as a highly efficient anion pump, i.e., arsenite transporter ATPase [21]. Deficiency of ARSB may cause skeletal abnormalities such as mucopolysaccharidosis type VI (MPS VI) disease, which occurs in humans and cats and is also called Maroteaux–Lamy syndrome, where the tissues and organs become enlarged, inflamed, or scarred [22,23].

Several novel genes necessary for quinone synthesis, including *Tcas-ql ARSB*, have been identified from the exocrine gland transcriptome in the red flour beetle [6]. Since lysosomes are the waste disposal system in the cell [24] and the regulatory mechanism of *ARSB* genes in insects has not been clarified yet, the proposed possible functions of ARSB proteins are as follows. Firstly, ARSBs may have important roles in the detoxication of toxic substances in secretory glands (negative feedback regulation), the silencing or knockdown of which leads to the inhibition of external secretions and attenuate external immunity [4,6,11]; secondly, ARSBs may not be located in lysosomes but in the cytoplasm or elsewhere, functioning as essential transporters for the intermediates involved in quinone synthesis (positive feedback control), which thus act as a key enzyme responsible for the activation of the newly translated transporters or other vital related proteins, or simply controlling the energy source of transportation, such as the pH gradient or ion donators [6,10,25].

It can be seen from the GenBank database that *ARSBs* have been isolated and characterized in a very limited number of insect species, such as *Aedes aegypti*, *T. castaneum*, *Nasonia vitripennis*, *Drosophila ananassae*, *Drosophila serrata*, *Acyrthosiphon pisum*, *Halyomorpha halys*, and *Bemisia tabaci* [6,26]. However, there is almost no information about whether these genes are involved in external immune defense. The red palm weevil (RPW), *Rhynchophorus ferrugineus* (Coleoptera: Curculionidae), is native to India and is the most notorious palm trunk-boring pest worldwide [27]. In China, RPW, as an invasive pest, has killed approximately 20,000 coconut trees, with an infestation area over 10,000 km^2^, and presents serious threats to the green ecological safety of coastal areas [28,29]. Although the effective biocontrol of RPW by pathogenic microbes has been validated under laboratory conditions [30,31], successful colonization by this species is partly due to strong individual immunity against exogenous substances [4]. Characterization of the immune reactions, particularly external defense, is required to elucidate the invasive mechanisms of RPW and the interaction between RPW and pathogens. When RPW larvae were infected by pathogens, obvious secretions with antimicrobial activity, such as p-benzoquinone, were released from the oral cavity [32]. In recent years, this phenomenon has also been found to be increasingly common in other insects [33,34]. Previously, a sialotranscriptome of adult female, *Culex tarsalis*, revealed that oral secretions (OS) were generally produced by salivary glands [35].

Therefore, in the present study, we hypothesize that the *ARSB* genes are expressed specifically and most highly in the salivary gland of RPW larvae; perhaps because of increased antimicrobial activity and p-benzoquinone concentration in OS produced by RPW larvae, due to a variation in the expression of such genes that further enhances external immune efficacy. In other words, ARSB may act as a regulator of external immunity by controlling the p-benzoquinone biosynthetic process in RPW larvae. To test these hypotheses, we intensively investigate and demonstrate the role of three novel *RfARSBs*, designated as *RfARSB-0311*, *RfARSB-11581*, and *RfARSB-14322*, in the p-benzoquinone synthesis of RPW larval OS involved in the external immune defense with comprehensive methods including full-length cDNA cloning, gene expression profiling in different tissues/organs (especially salivary glands), RNA interference (RNAi), functional assays, and targeted metabonomic sequencing techniques.

## 2. Results

### 2.1. Sequence Characteristics and Phylogenetic Analysis of RfARSBs

The full-length complete cDNA sequences of three *RfARSBs*, namely, *RfARSB-0311* (GenBank accession number: MN938355), *RfARSB-11581* (GenBank accession number: MN938356), and *RfARSB-14322* (GenBank accession number: MN938357), possessing 2112 bp (Figure S1), 1814 bp (Figure S2), and 1758 bp (Figure S3), respectively, were obtained by 5′ and 3′ RACE-PCR amplification on the basis of the putative conserved domain of ARSB from *R. ferrugineus* transcriptome data. The open reading frames (ORFs) of *RfARSB-0311*, *RfARSB-11581*, and *RfARSB-14322* encoded 630 (Figure S1), 535 (Figure S2), and 522 (Figure S3) amino acid residues, respectively. The RfARSBs, a class of secreted proteins, were composed of signal peptides with the first 18, 18, and 21 residues and core functional domains constituting sulfatase and phosphodiesterase, respectively (Figures S1–S3), which confirmed that these proteins could be categorized as alkaline phosphatase and sulfatase superfamily members of arylsulfatase. However, it was shown that only RfARSB-0311 possessed the transmembrane region (Figure S1). Both RfARSB-0311 and RfARSB-14322 had five putative N-linked glycosylation sites (Figure S1 and Figure S3), but only four sites for RfARSB-11581 were found (Figure S2). In addition, there were also some potential O-linked glycosylation sites in RfARSB-0311 (seven sites), RfARSB-11581 (four sites), and RfARSB-14322 (five sites) (Figures S1–S3).

**Figure 1:**
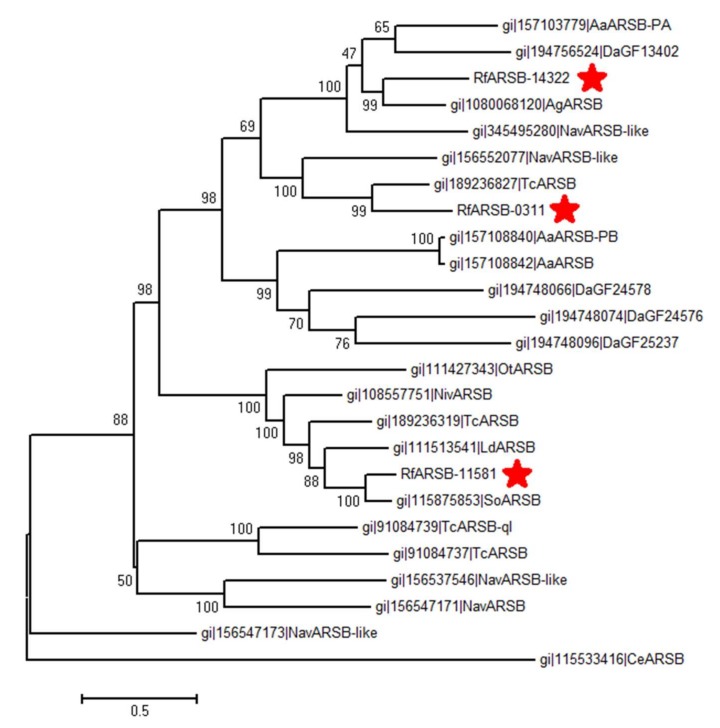
Phylogenetic tree of homologs of the three novel arylsulfatase B (ARSB) proteins from *R. ferrugineus* with other known insect and nematode ARSBs. The amino acid sequences of the complete proteins were aligned to construct the dendrogram using the maximum likelihood (ML) method. Bootstrap values shown next to nodes were based on 1000 repetitions. The scale on the bottom of the dendrogram shows the degree of dissimilarity. The abbreviations of species names and accession numbers of the corresponding protein sequences in GenBank are as follows: Aa, *Aedes aegypt* (XP_001648126.1; XP_001650408.1; XP_001650409.1); Ag, *Anoplophora glabripennis* (XP_018576345.1); Ce, *Caenorhabditis elegans* (NP_001041231.1); Da, *Drosophila ananassae* (XP_001956470.1; XP_001956474.1; XP_001956485.1; XP_001960527.1); Ld, *Leptinotarsa decemlineata* (XP_023025565.1); Nav, *Nasonia vitripennis* (XP_001607560.1; XP_001603886.1; XP_001603910.1; XP_001604760.1; XP_001606377.2); Niv, *Nicrophorus vespilloides* (XP_017769891.1); Ot, *Onthophagus Taurus* (XP_022918215.1); Rf, *Rhynchophorus ferrugineus*; So, *Sitophilus oryzae* (XP_030747249.1); Tc, *Tribolium castaneum* (NP_001280526.1; XP_970917.1; XP_975218.2; XP_972832.2). RfARSBs characterized in this study are highlighted with red asterisks.

To further uncover the evolutionary relationship between RfARSBs and other invertebrate ARSBs, 22 related ARSB protein sequences with relatively high similarity and homology from several species, including *A. aegypti*, *Anoplophora glabripennis*, *Caenorhabditis elegans*, *D. ananassae*, *Leptinotarsa decemlineata*, *N. vitripennis*, *Nicrophorus vespilloides*, *Onthophagus taurus*, *Sitophilus oryzae*, and *T. castaneum* were chosen to construct the phylogenetic tree. The dendrogram showed that all 25 ARSBs were divided into two main clades (Figure 1). Furthermore, it was found that RfARSB-0311, RfARSB-11581 and RfARSB-14322 were clustered into three subgroups, similar to the division of AaARSBs, DaARSBs, TcARSBs, and NavARSBs, but they all had the closest kinship with ARSB proteins of beetles, TcARSB, SoARSB, and AgARSB, respectively (Figure 1). As expected, CeARSB formed a single branch that excluded insect ARSBs (Figure 1). This finding indicates that the three kinds of ARSBs in RPW not only evolved conservatively in Diptera, Coleoptera, and Hymenoptera but also had a closer evolutionary relationship to coleopteran arylsulfatases than to those of other orders, reflecting that they may signify the division in functions.

### 2.2. Tissue/Organ Expression Profiles of RfARSBs

**Figure 2:**
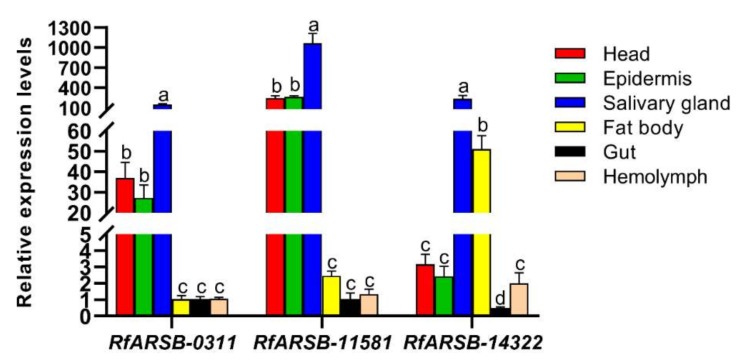
Gene expression profiles of *RfARSBs* across different tissues/organs in *R. ferrugineus* larvae. Expression levels were normalized to the *GAPDH* reference gene. Data are shown as the mean ± standard error (SE) from five independent biological repetitions. Different lowercase letters above the bar indicate statistically significant differences among different tissues/organs for the same gene at *p* < 0.05 (one-way analysis of variance (ANOVA) followed by Tukey’s honestly significant difference (HSD) multiple comparisons).

To investigate and compare the expression patterns of the three *RfARSBs* under normal circumstances in the absence of infection, we analyzed their relative expression in different tissues/organs of RPW larvae. *RfARSBs* were expressed in all the tested tissues/organs, including the head, epidermis, salivary gland, fat body, gut and hemolymph. Significant differences were further detected in the transcript levels of *RfARSBs* across different tissues/organs (*RfARSB-0311*: *F*_5,24_ = 55.877, *p* < 0.001; *RfARSB-11581*: *F*_5,24_ = 44.564, *p* < 0.001; *RfARSB-14322*: *F*_5,24_ = 20.125, *p* < 0.001; Figure 2). The lowest abundances of *RfARSB-0311* and *RfARSB-11581* were observed in the fat body, gut, and hemolymph, while *RfARSB-14322* was barely expressed except in the salivary gland and fat body (Figure 2). However, the results showed that the three *RfARSBs* were all expressed specifically and most highly in the salivary gland, the expression in which was at least three times, three times, and four times higher, respectively, than that in other tissues/organs (Figure 2). The abundant transcripts of *RfARSBs* in the exocrine gland suggested that *RfARSBs* were probably involved in the external immune defense of this pest.

### 2.3. Antimicrobial Activity of Larval Oral Secretions Induced by M. anisopliae Infection

**Figure 3:**
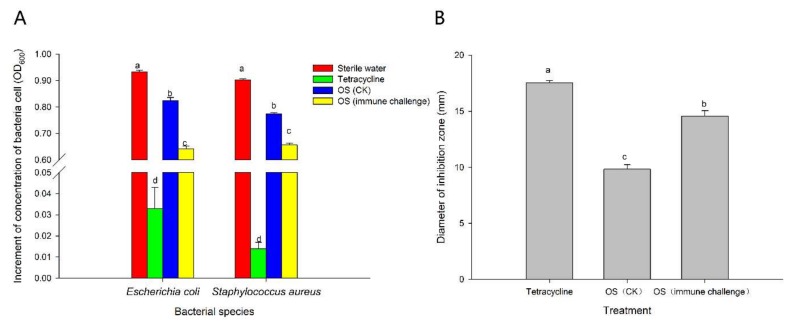
Microbial growth inhibition assays of oral secretions on (**A**) bacteria and (**B**) fungi in red palm weevil (RPW) larvae after immune challenge with *Metarhizium anisopliae*. Data are shown as the mean ± SE from ten independent biological repetitions. Different lowercase letters above the bar indicate statistically significant differences among different treatments for the same indicator microbe at *p* < 0.05 (one-way ANOVA followed by Tukey’s HSD multiple comparisons).

In our experiments, a gram-negative bacterium (*E. coli*), a gram-positive bacterium (*S. aureus*), and a fungus (*M. anisopliae*) were used to test the strength of the external immune defense mediated by weevil glands. Obviously, OS produced by RPW larvae can inhibit the growth of several microbes (Figure 3). However, when larvae were exposed to *M. anisopliae*, the antibacterial activity of OS against *E. coli* and *S. aureus* was more dominant than that observed in individuals treated with 0.05% Tween 80, reducing the average OD_600_ increment by 22.09% and 15.25%, respectively (inhibition of *E. coli*: *F*_3,36_ = 1626.102, *p* < 0.001; inhibition of *S. aureus*: *F*_3,36_ = 6351.686, *p* < 0.001; Figure 3A). Similarly, the diameter of the inhibition zone against *M. anisopliae* also significantly increased from 9.83 ± 0.41 mm to 14.59 ± 0.47 mm compared with the control group (*F*_2,27_ = 104.374, *p* < 0.001; Figure 3B).

### 2.4. Contents of p-Benzoquinone in Oral Secretions Produced by Larvae Following Infection with M. anisopliae

**Figure 4:**
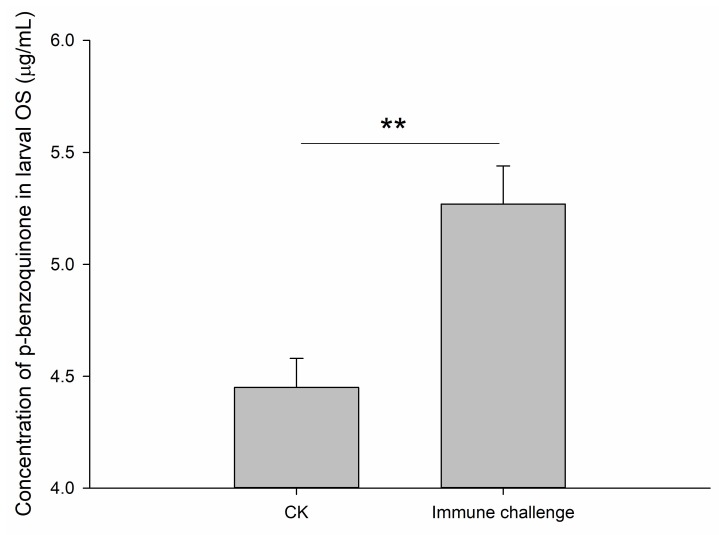
Quantification of p-benzoquinone chemical by gas chromatography-mass spectrometry (GC-MS) in oral secretions of RPW larvae after immune challenge with *Metarhizium anisopliae*. Data are shown as the mean ± SE from six independent biological repetitions. The asterisks marking the Student’s *t* test result indicate that there is a significant difference between the two treatments (**, *p* < 0.01).

Analysis of targeted metabonomics by gas chromatography-mass spectrometry (GC-MS) showed a significant difference in the concentration of p-benzoquinone secreted by the RPW larval oral cavity between the control (average of 4.45 µg/mL) and *M. anisopliae* treatments (average of 5.27 µg/mL) (*t*_10_ = −3.813, *p* = 0.003; Figure 4). Therefore, not only the inhibitory efficiency of larval OS, but also the quantity of p-benzoquinone present in these secretions was enhanced after immune challenge.

### 2.5. Expression Levels of RfARSBs after Immune Challenge

**Figure 5:**
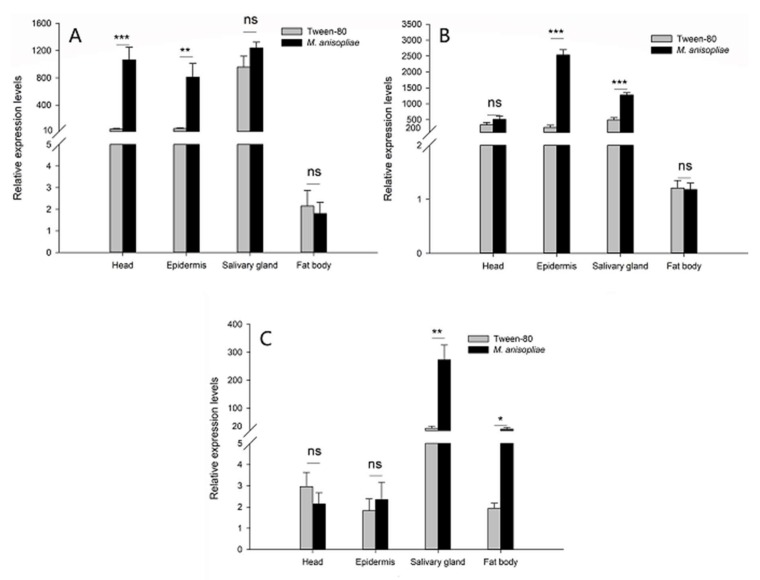
Analysis of the expression levels of (**A**) *RfARSB-0311*, (**B**) *RfARSB-11581*, and (**C**) *RfARSB-14322* in the head, epidermis, salivary gland, and fat body after immune challenge with *Metarhizium anisopliae*. Expression levels were normalized to the *GAPDH* reference gene. Data are shown as the mean ± SE from five independent biological repetitions. The asterisks marking the Student’s *t* test results indicate that there is a significant difference between the two treatments (*, *p* < 0.05; **, *p* < 0.01; ***, *p* < 0.001), while “ns” indicates that there is no significant difference between the control and treatment groups.

To determine the potential roles of *ARSBs* in RPW larval immunity in vitro, their transcriptional response to external infections with microbial elicitors was examined. As a result of the relatively high mRNA abundance in the head, epidermis, salivary gland, and fat body (Figure 2), we detected changes in the expression profiles of *RfARSBs* only in these four tissues/organs, excluding the gut and hemolymph, after *M. anisopliae* challenge. Although the mRNA abundance of *RfARSB-0311* in the head (*t*_8_ = −5.493, *p* < 0.001) and epidermis (*t*_8_ = −3.758, *p* = 0.006) increased dramatically after *M. anisopliae* challenge compared with 0.05% Tween 80 treatment, no significant difference was observed in the fat body (*t*_8_ = 0.392, *p* = 0.705) (Figure 5A). Transcript expression of *RfARSB-11581* in the head was not affected by immune challenge with *M. anisopliae*, unlike the expression of *RfARSB-14322* (head: *t*_8_ = −1.329, *p* = 0.220; epidermis: *t*_8_ = −12.612, *p* < 0.001; fat body: *t*_8_ = 0.143, *p* = 0.890; Figure 5B). However, infection with *M. anisopliae* could potentially induce the expression of *RfARSB-14322* in the fat body (*t*_8_ = −3.955, *p* = 0.017), while the microbial elicitor did not enhance the abundance of this transcript in the head (*t*_8_ = 0.960, *p* = 0.365) and epidermis (*t*_8_ = −0.519, *p* = 0.618) (Figure 5C). Interestingly, in salivary glands, the main synthetic and secretory sites of larval OS containing p-benzoquinone, *M. anisopliae* challenge sharply increased the relative expression level of *RfARSB-11581* (*t*_8_ = −7.763, *p* < 0.001; Figure 5B) and *RfARSB-14322* (*t*_8_ = −4.557, *p* = 0.002; Figure 5C) by 159.25% and 851.06%, respectively, but not *RfARSB-0311* (*t*_8_ = −1.571, *p* = 0.155; Figure 5A). Altogether, these results indicate that *RfARSBs* in the head, epidermis, salivary gland, and fat body can be induced by immune challenge.

### 2.6. Roles of RfARSBs in External Immunity

To further explore the possible roles of RfARSBs in mediating the biosynthesis of p-benzoquinone involved in external immunity, the immunosuppressive efficiency and the amount of p-benzoquinone in larval OS were investigated and compared based on RNAi.

**Figure 6:**
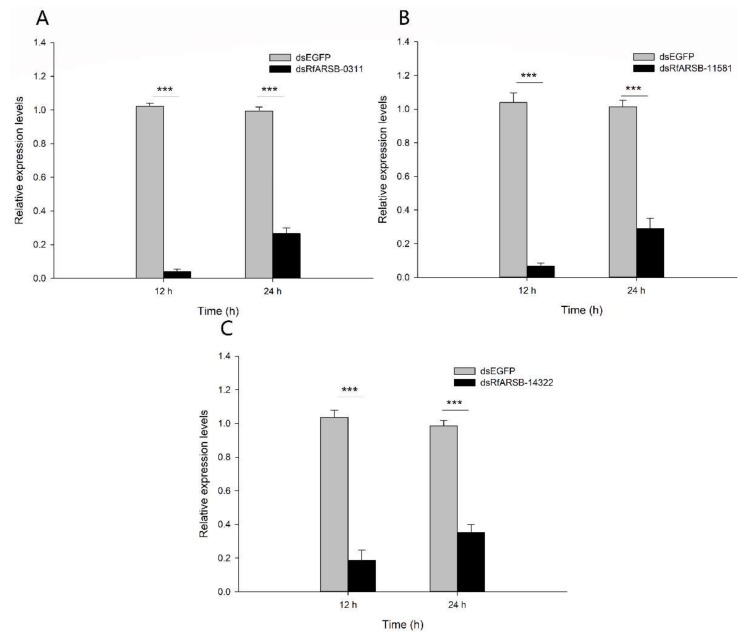
Determination of larval RNAi efficiency of (**A**) *RfARSB-0311*, (**B**) *RfARSB-11581*, and (**C**) *RfARSB-14322* detected at 12 h and 24 h after dsRNA injection. Expression levels were normalized to the *GAPDH* reference gene and the dsEGFP group. Data are shown as the mean ± SE from five independent biological repetitions. The asterisks marking the Student’s *t* test results indicate that there is a significant difference between the dsEGFP and dsRfARSBs treatments (***, *p* < 0.001).

Double-stranded RNA (dsRNA) of *RfARSBs* was injected into larvae for target gene silencing, and the RNAi efficiency at 12 h and 24 h was then detected. The results revealed that the delivery of dsRNA into the body cavity of RPW larvae could significantly knock down the expression of *RfARSBs*. The dsRNA delivery caused significant downregulation of the expression of *RfARSB-0311*, *RfARSB-11581* and *RfARSB-14322* at 12 and 24 h post injection. The maximum RNAi efficiencies were observed at 12 h post dsRNA injection, where the transcript abundance of *RfARSB-0311*, *RfARSB-11581* and *RfARSB-14322* decreased by 95.98% (*t*_8_ = 42.250, *p* < 0.001; Figure 6A), 93.46% (*t*_8_ = 18.081, *p* < 0.001; Figure 6B) and 81.95% (*t*_8_ = 16.085, *p* < 0.001; Figure 6C), respectively. Whereas, at later time point (24 h), the observed downregulations of *RfARSB-0311*, *RfARSB-11581* and *RfARSB-14322* were only 73.24% (*t*_8_ = 9.857, *p* < 0.001; Figure 6A), 71.47% (*t*_8_ = 11.220, *p* < 0.001; Figure 6B) and 64.34% (*t*_8_ = 11.010, *p* < 0.001; Figure 6C), respectively. Thus, the time point of 12 h post injection of dsRfARSBs was selected to perform the following experiments.

**Figure 7:**
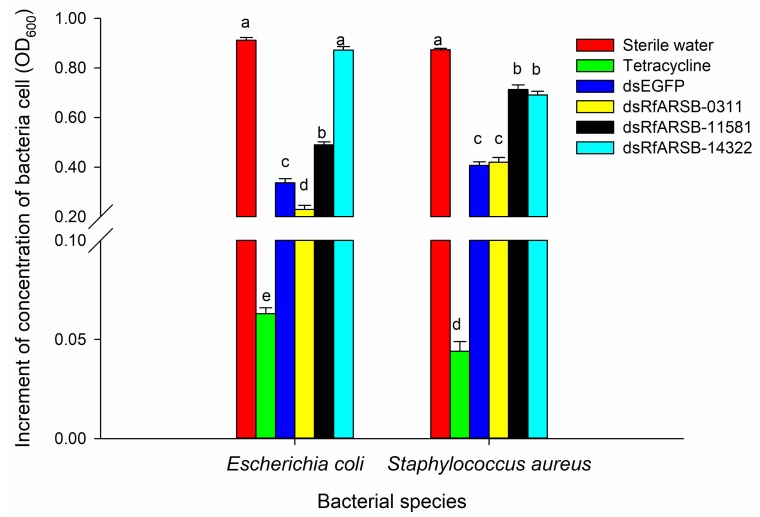
Effect of *RfARSBs* silencing on the antibacterial activity of oral secretions in RPW larvae at 12 h after dsRNA injection. Data are shown as the mean ± SE from ten independent biological repetitions. Different lowercase letters above the bar indicate statistically significant differences among different treatments for the same indicator bacterial species at *p* < 0.05 (one-way ANOVA followed by Tukey’s HSD multiple comparisons).

**Figure 8:**
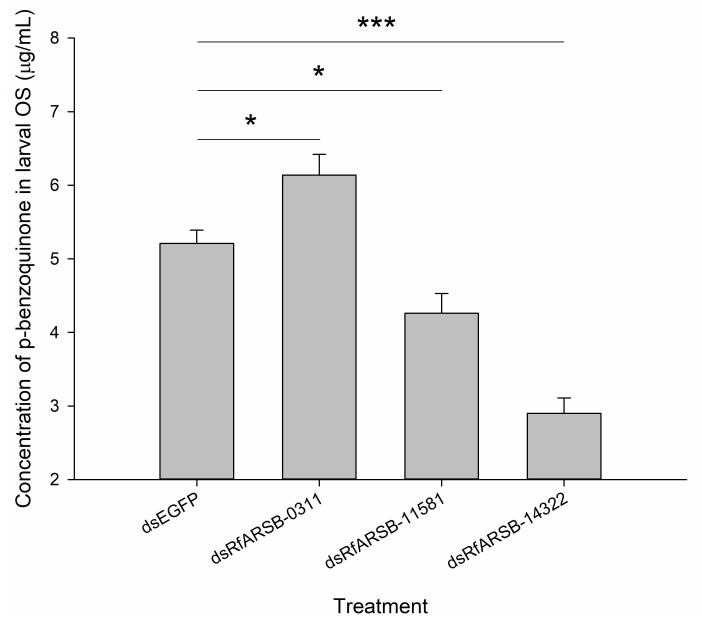
Effect of *RfARSBs* silencing on the quantity of p-benzoquinone in oral secretions produced by RPW larvae at 12 h after dsRNA injection. Data are shown as the mean ± SE from six independent biological repetitions. The asterisks marking the Student’s *t* test results indicate that there is a significant difference between the treatment and the control group (*, *p* < 0.05; ***, *p* < 0.001).

The results of bacterial inhibition assays evidently demonstrate that silencing of *ARSB*-related genes expressed in RPW larvae could alter the antibacterial activity of the OS, thereby affecting external immunity (Figure 7). As expected, *RfARSB-11581* and *RfARSB-14322* knockdown both led to a notable decline in inhibitory efficiency against *E. coli* (*F*_5,54_ = 659.182, *p* < 0.001) and *S. aureus* (*F*_2,54_ = 434.716, *p* < 0.001) (Figure 7). Surprisingly, we found that the antibacterial activity against *S. aureus* was not affected by silencing *RfARSB-0311*, while the OS, upon knockdown of this gene, instead inhibited the growth of *E. coli* (Figure 7). Moreover, the concentration of p-benzoquinone in larval OS decreased significantly by 18.23% and 44.34% after injection of dsRfARSB-11581 (*t*_10_ = 2.916, *p* = 0.015) and dsRfARSB-14322 (*t*_8_ = 8.219, *p* < 0.001), respectively, compared with that in the enhanced green fluorescent protein (EGFP) dsRNA (dsEGFP) group (Figure 8), which further indicates a role for RfARSB-11581 and RfARSB-14322 in mediating the biosynthesis of p-benzoquinone involved in external immune defense. Interestingly, a low-grade increase in p-benzoquinone concentration from 5.21 to 6.14 µg/mL was observed when *RfARSB-0311* was silenced (*t*_8_ = −2.760, *p* = 0.020; Figure 8), implying that RfARSB-0311 might be involved in the degradation of p-benzoquinone, thus reducing the external immune inhibitory efficiency. Together, these results reveal that RfARSBs can affect the external immunity of RPW larvae by regulating and controlling the level of p-benzoquinone in OS.

## 3. Discussion

Sulfatases, which represent a large protein family, are involved in hormone biosynthesis, cellular signal regulation, and even degradation of macromolecules [14,36]. They have the ability to hydrolyze sulfates from different sulfated substrates, such as steroids, carbohydrates, proteoglycans and glycolipids [13]. All the sulfatases described in previous studies contain the Ca-formylglycine (fgly) residue, which is essential for enzyme activity, at their catalytic sites [12,16]. In addition to the core sequence C/S-X-P-X-R being relatively well conserved in such enzymes [15,17], they also possess signal peptides that target them to the endoplasmic reticulum [13]. Accordingly, the three novel ARSB proteins we characterized in RPW were all members of the alkaline phosphatase and sulfatase superfamily. The bioinformatic analysis indicated that their active amino acid sites were conserved, and signal peptide regions could be predicted, illustrating the similar general structures and characteristics of sulfatases.

The ARSB protein existing in *E. coli* as the ion transporter superfamily member possesses 12 transmembrane segments and membrane topologies similar to those of many carrier proteins [18,19], functioning as a catalyst for reverse transport of metal-proton exchange [20]. In addition, ARSB is also regarded as a membrane protein in *S. aureus* that efficiently transports ATP [21]. Likewise, we also found that only ARSB-0311 contained a region of the transmembrane domain in the novel ARSBs of RPW, suggesting that it may act as a transporting ATPase.

There were few differences in the structure and characteristics of ARSBs among different species after the ARSB amino acid sequences of RPW were compared with those of other known insects, including *A. aegypti*, *A. glabripennis*, *D. ananassae*, *L. decemlineata*, *N. vitripennis*, *N. vespilloides*, *O. Taurus*, *S. oryzae*, and *T. castaneum*. Meanwhile, ARSBs have been revealed to have multiple conserved regions, such as Ca^2+^- and substrate-binding sites. This result was basically consistent with the report of Li et al. (2013) on *T. castaneum*, a typical Coleoptera model insect [6]. Cysteine generally forms a disulfide bond (S–S) to stabilize protein configuration or is involved in the formation of enzymatically active centers [12,15]. However, we surprisingly found that the cysteine at the Ca^2+^-binding site of RfARSB-11581 was mutated to serine, which may affect the function of this protein.

Although the number of sulfatase-related genes varies from 1 to 100 in different species, most insects usually possess only one to three [13]. For example, there are eight kinds of sulfatases in total in fruit flies, but only three different sulfatase genes exist in *C. elegans* and one in the *B. tabaci* Q biotype [6,26]. In the present study, we also identified only three genes in RPW. It was seen from the phylogenetic tree of ARSBs that the *RfARSBs* had the closest kinship with such genes in another model beetle, *T. castaneum* (*TcARSBs*), in general, while interestingly, there was also a difference in the evolutionary relationship among the three *RfARSBs*. Therefore, different evolutionary directions may appear among *RfARSB-0311*, *RfARSB-11581*, and *RfARSB-14322*. In *T. castaneum*, the *ARSB* gene showing the quinone-less phenotype was highly expressed in secretory glandular cells, which confirmed that it can regulate external immune chemical defense by synthesizing quinones in secretions produced by odoriferous defensive stink glands [6]. However, it seems that other *ARSB* genes in red flour beetles did not play a role in the biosynthesis of quinones [6], possibly caused by the different evolutionary directions of *ARSBs*.

Previous studies have definitively shown that OS in RPW larvae are capable of inhibiting the growth of microbes in vitro and that p-benzoquinone responsible for external immunity is a major active component among all secretory chemicals [32]. This phenomenon is not unique to RPW and has also been confirmed in many other insects, such as *T. castaneum* [3], *T. molitor* [8], *Nicrophorus vespilloide* [37], and *Helicoverpa armigera* [34]. OS are produced by salivary glands [35]. In the present study, we correspondingly detected the specific overexpression of all *RfARSBs* in salivary glands, the mRNA abundance of which was much higher than that in other tissues/organs, including the head, epidermis, fat body, gut, and hemolymph. This result preliminarily implied that *RfARSBs* were related to the regulation of p-benzoquinone. However, the specific functions of *RfARSB-0311*, *RfARSB-11581*, and *RfARSB-14322* may be predicted as being different because of the diversity of their genetic relationships.

Specific chemical compounds in external immune secretions, including metabolites or excreta from insects, will help to protect individuals from being attacked by natural enemies, especially pathogens [4,38]. Tenebrionid beetles actively use quinones produced for external immune defense to reduce or manipulate microbial pressure in the environment and to keep their food source from spoiling [39,40]. For the purpose of intruding into insects successfully, *Metarhizium* can form special structures, including appressoria, and then secrete a variety of suitable enzymes (such as chitinase) to destroy the cuticle of the host [41]. It is inevitable that these organisms have to overcome the inhibitory activity of some substances from the body surface and a series of immune responses from hosts [42]. *M. anisopliae*, as an important fungus widely used in the biocontrol of pests, has been proven to be the pathogen of RPW, reducing the survival of certain larvae [43]. As expected, not only the external immunosuppressive efficacy of OS but also the synthesis of multiple antimicrobial compounds in OS, especially the concentration of p-benzoquinone, increased markedly after RPW larvae were infected by *M. anisopliae*, suggesting that external immunity could be induced by microbial elicitors. In addition, we found that the expression of immune-related genes in different tissues/organs also changed significantly. The transcript levels of both *RfARSB-11581* and *RfARSB-14322* increased in the salivary gland. In addition to the salivary gland, the mRNA abundance of *RfARSB-11581* was upregulated in only the epidermis, while an upward trend was observed in the fat body for *RfARSB-14322*. Interestingly, there was a dramatic increase in the expression of only *RfARSB-0311* in the head. However, this phenomenon happened to *RfARSB-0311* and seems to share some similarities with the report in *B. tabaci* regarding to *ARSB* gene [26]. The salivary gland located in the head and thorax opens in the oral cavity [26], so there may also be some connection between the head and individual external immune response, perhaps resulting in an increased immune-related gene expression level in the head after infection. On the other hand, after insects are challenged with *M. anisopliae*, the central nervous system is affected and activated in addition to the immune system [42]. We accordingly speculate that unlike *RfARSB-11581* and *RfARSB-14322*, the *RfARSB-0311* gene may also play a potential role in the nervous response of RPW to pathogens and be involved in the regulation of the nervous system. Even so, the specific reasons need to be further explored. The precursor of p-benzoquinone is the metabolite of the tanning and hardening pathway for the ectoskeleton and epidermis, so insects generally exhibit enhanced tanning and hardening of the cuticle when stressed by pathogens [44]. Thus, it was indicated from these results that although the variations of these three *RfARSB* expression in different tissues/organs after RPW larvae that were challenged with pathogens were inconsistent, all of them might have the same substrate and may further play a regulatory role in the biosynthesis of p-benzoquinone in OS.

For quinone biosynthesis, it has been reported in another quinone-producing tenebrionid beetle, *E. longicollis*, that alkylated benzoquinones are formed by acetate condensation, whereas p-benzoquinone is generated from preformed aromatic rings of amino acids, including tyrosine and phenylalanine [9]. In glandular secretory cells, p-quinones are present in the form of phenolic β-glucosides contained in the apical part, which are then transferred to the inner part of the gland and form active quinones via a series of enzymatic reactions [10]. Thus, it can be inferred that arylsulfatase must play an irreplaceable role in the metabolism of p-benzoquinone.

Presently, because mounting evidence has confirmed that quinones play an important role in modulating external immunity [3,6,32,38], we undoubtedly associate the amount of p-benzoquinone with the antimicrobial activity of OS. The relatively high abundance of *RfARSB* transcripts in the salivary gland and their immunological roles against pathogens suggested that these genes might affect external immunity, which led us to determine whether they were involved in regulating p-benzoquinone synthesis in OS produced by RPW larvae using RNAi. Our data demonstrate that silencing of *RfARSB-11581* or *RfARSB-14322* dramatically reduced the antibacterial activity of larval OS, and the p-benzoquinone content decreased as well. Therefore, it was revealed that the synthesis of p-benzoquinone would be positively promoted by the presence of *RfARSB-11581* and *RfARSB-14322*, resulting in the enhancement of external immunity. This finding is similar to that obtained for *T. castaneum*. Specifically, the knockdown of *Tcas-ql ARSB* resulted in the absence of quinones and a decrease in antimicrobial activity produced by glandular secretions [6].

Interestingly, we surprisingly observed a contrasting phenomenon. After dsRfARSB-0311 was injected into RPW larvae, an ascending trend was observed for the antibacterial activity of larval OS and the content of p-benzoquinone. This result reveals that *RfARSB-0311* may play a negative regulatory role in the synthesis of p-benzoquinone. It is known that p-benzoquinone is highly reactive, unstable, and toxic. In addition to immune defense, insects also use this compound as a tanning agent and to sclerotize cuticles, indicating the need for perfect handling and detoxication systems [4,6]. Therefore, *RfARSB-0311* may exactly provide an autodetoxication mechanism.

In summary, external immune responses, including OS in the chemical defense system, endow insects with the potential ability to defend themselves against pathogens [4,38]. Therefore, we are very interested in checking how external immunity is affected when the chemical defense system is knocked down. Conclusively, we cloned and characterized three novel RfARSBs from the invasive pest *R. ferrugineus* larvae and revealed their differential biological properties in external immunity for the first time. The three arylsulfatases in RPW had conserved relationships in insects and possessed novel sequence features and functional characteristics, which led to a functional division in external immunity. Our data demonstrate that RfARSBs acted as modulators to achieve a balance between the quantity of p-benzoquinone in larval OS and external resistance to pathogens. In this regulatory process, RfARSB-11581 and RfARSB-14322 functioned in the biosynthesis of p-benzoquinone mediating external immune defense, while RfARSB-0311 appeared to have a negative regulatory role. These explorations could further provide information on the functional diversification of insect ARSBs and have paved the way for the elucidation of external immunity in RPW, helping reveal its invasive mechanism and the interactions between RPW and pathogens.

## 4. Materials and Methods

### 4.1. Collection and Rearing of Insects

Different life stages of red palm weevil (RPW), *Rhynchophorus ferrugineus*, including mature larvae, pupae, and adults, were collected from infested date palm trees in Zhangzhou city (117.62° E, 24.13° N) and Fuzhou city (119.78° E, 25.52° N) in Fujian Province during the years 2016–2018. In the laboratory, the larvae were fed separately with fresh sugarcane, *Saccharum officinarum*, in climatic chambers at 25 ± 1 °C, 75% relative humidity (RH), and a photoperiod of 24 h darkness, while adults were maintained under the above conditions except with a 12:12 h (light: dark) photoperiod. The diets were replaced every 7 days. The seventh-instar larvae (weight, approximately 3 g) were used for the following experiments.

### 4.2. Microbial Strains and Cultures

The pathogen responsible for external immune challenges, the *Metarhizium anisopliae* var. *anisopliae* strain, was isolated and obtained from RPW cadavers that died of natural disease. This strain was deposited under accession number MF467274 in GenBank. Briefly, the *M. anisopliae* strain was cultured in Petri dishes containing potato dextrose agar (PDA) medium (Shanghai Bio-way Technology Co., Ltd., Shanghai, China) at 25 °C until a large number of dark green conidia were obtained. Viable germinating conidia suspended in 0.05% Tween 80 were then counted using a hemocytometer.

In addition to *M. anisopliae*, two other bacterial strains, namely, *E. coli* DH5α (Beijing TransGen Biotech Co., Ltd., Beijing, China) and *S. aureus* (Nanjing Biotechnology Co., Ltd., Nanjing, China), were also used in microbial inhibition assays. Bacterial cultures were grown in Luria-Bertani (LB) liquid medium (tryptone 10 g, NaCl 10 g, yeast extract 5 g, distilled water 1000 mL) at 37 °C and 200 r/min until the optical density at 600 nm wavelength (OD_600_) reached 0.6.

### 4.3. Immune Challenges in Vitro

Sugarcane used to feed larvae was uniformly covered with 1 mL of 1.0 × 10^5^ conidia /mL *M. anisopliae* suspensions containing 0.05% Tween 80. Because the spores of *Metarhizium* usually germinate 24 h after inoculation [45], larvae that had been starved for 24 h previously were exposed to fungal conidia in solution by allowing them to come in contact with the conidia in vitro and feed freely for 24 h. The control individuals were treated with 0.05% Tween 80 solution. We subsequently determined the following three indexes and parameters: (1) the antimicrobial activity of OS against *E. coli*, *S. aureus*, and *M. anisopliae*; (2) the concentration of p-benzoquinone in OS; and (3) the relative expression of *ARSB* genes in different tissues/organs.

### 4.4. Collection of Larval Oral Secretions

We followed and referred to the method described by Turlings et al. (1993) and Chen et al. (2019) to collect larval OS [34,46]. Larvae were gently fixed between fingers and thumb and would usually spit out OS under extrusion stimulus. A 10-µL pipette tip was used to softly touch the larvae at the mouth cavity. The OS samples were then collected into 1.5-mL Eppendorf tubes and stored at −80 °C to be further used in microbial inhibition assays and for the quantification of p-benzoquinone. Each sample contained at least 9 larvae.

### 4.5. Amplification, Purification, and Analysis of Full-Length cDNA Sequences of RfARSB Genes

Red flour beetle ARSB protein, involved in the biosynthesis of quinones produced by exocrine glands, was used to initially search the protein database of the National Center for Biotechnology Information (NCBI) (http://www.ncbi.nlm.nih.gov/protein/). Then, three *ARSB* homologous fragments were selected and obtained from the transcriptome data for RPW [47] with the BLAST algorithm using BioEdit 7.0.5 software [48].

Total RNA was extracted from the whole-body homogenates of RPW larvae by TRIzol reagent (Invitrogen, Carlsbad, CA, USA) according to the manufacturer’s instructions. Electrophoresis on a 1% agarose gel and a NanoDrop 2000 ultra-micro spectrophotometer (Thermo Fisher Scientific Inc., Waltham, MA, USA) was performed to determine the quality, integrity, and concentration of the extracted RNA. cDNA was generated from 1 µg of total RNA using the PrimeScript^®^ First Strand cDNA Synthesis Kit (Takara Biotechnology Co., Ltd., Dalian, China) with PrimeScript RTase as reverse transcriptase by incubating at 42 °C for 15 min followed by 85 °C for 15 s in a total reaction volume of 20 µL, where the DNase I was additionally used to remove any potential genomic DNA (gDNA) contamination before reverse transcription (RT). Then, polymerase chain reaction (PCR) was further performed (25-µL reaction system) using the 2 × Taq Plus MasterMix Kit (Tiangen Biotech Co., Ltd., Beijing, China) under the following conditions: initial denaturation at 94 °C for 3 min; 35 cycles of denaturation at 94 °C for 35 s, annealing at 53 °C for 30 s, and extension at 72 °C for 1 min; and a final extension at 72 °C for 10 min. The obtained PCR products purified by 1% agarose gel electrophoresis were cloned and ligated into the pEASY^®^-T1 vector (TransGen Biotech, Co., Ltd., Beijing, China). The positive colonies were selected for sequencing by Invitrogen Trading (Shanghai) Co., Ltd., Shanghai, China. The accession numbers deposited in GenBank were MN938355–MN938357. Touchdown and nested PCR approaches were employed for rapid amplification of the 3′ and 5′ cDNA ends (RACE) of the three *RfARSBs* following the protocol for the SMARTer^TM^ RACE cDNA Amplification Kit (Takara Biotechnology Co., Ltd., Dalian, China). The subcloning and sequencing of the RACE-PCR products were conducted as described above. All specific primers (Table S1) were designed using Primer Premier 5.0 and synthesized by Sangon Biotech (Shanghai) Co., Ltd., Shanghai, China.

Full-length cDNA sequences of the three *RfARSBs* were analyzed with the DNAMAN 9.0 program and SMART tool (http://smart.embl-heidelberg.de/) to deduce amino acid sequences and predict the functional domains of the encoded proteins, respectively. The signal peptides and transmembrane domains from amino acid sequences were detected using the SignalP 5.0 server (http://www.cbs.dtu.dk/services/SignalP/) and TMHMM 2.0 server (http://www.cbs.dtu.dk/services/TMHMM/), respectively. The putative O-linked and N-linked glycosylation sites were further checked through the NetOGlyc 4.0 server (http://www.cbs.dtu.dk/services/NetOGlyc/) and NetNGlyc 1.0 server (http://www.cbs.dtu.dk/services/NetNGlyc/), respectively. The obtained proteins were characterized on the basis of the conserved domains (CDD of NCBI, http://www.ncbi.nlm.nih.gov/Structure/cdd/wrpsb.cgi). The similarity and homology of the RfARSBs with ARSBs of other insects were compared with the NCBI Translated BLAST tool (https://blast.ncbi.nlm.nih.gov/Blast.cgi). *C. elegans* was selected as the out-group. CLUSTAL X 2.0 software was used for multiple sequence alignments of ARSBs from RPW and other invertebrates [49]. The phylogenetic tree was constructed on the basis of the maximum likelihood (ML) method with 1000 bootstrap repetitions using MEGA 5.2 [50]. All sequences were acquired from GenBank.

### 4.6. Evaluation of Gene Expression Profiles by Real-Time Quantitative PCR

To determine tissue/organ-specific expression profiles and the relative transcript levels of the three *RfARSBs*, different tissues/organs from RPW larvae, including head (excluding salivary gland), epidermis, fat body, gut, hemolymph, and salivary gland, were dissected in phosphate buffer saline (PBS: 8 g NaCl, 0.2 g KCl, 1.42 g Na_2_PHO_4_, 0.27 g KH_2_PO_4_, 1 L deionized water, pH 7.4) for subsequent total RNA extraction and preparation of cDNA following the method described above. Each sample contained at least 3 larvae. Real-time quantitative PCR (RT-qPCR) was performed using FastStart Universal SYBR Green Master supplemented with Rox (Roche, Basel, Switzerland) with a 20-µL reaction volume according to a previously described protocol [29]. The no-RT control was also prepared with 5× TransScript^®^ All-in-One No-RT Control SuperMix (Beijing TransGen Biotech Co., Ltd., Beijing, China) following the manufacturer’s instruction. For every tissue/organ sample, sterilized deionized water was used to substitute for the cDNA template as a negative control to detect whether there were other contaminations including unwanted gDNA. The standard curve for each tested gene was established through serial dilution (5×) of the cDNA template to ensure that the amplification efficiency of the primers was between 90% and 110%. The 2^−ΔΔ*C*t^ method was used to normalize and calculate gene expression levels using the software accompanying the ABI 7500 system (Invitrogen Trading (Shanghai) Co., Ltd., Shanghai, China) with glyceraldehyde-3-phosphate dehydrogenase (*GADPH*) as an internal reference gene [51]. All primer sequences are provided in Table S1.

### 4.7. RNA Interference

Targeted DNA fragments of *RfARSB-0311*, *RfARSB-11581*, and *RfARSB-14322* were amplified using specific primers (Table S1) conjugated to 20 bases of the T7 RNA polymerase promoter sequences. Then, dsRNA was generated by the MEGAscript^®^ RNAi Kit (Thermo Fisher Scientific Inc., Waltham, MA, USA) according to the manufacturer’s protocol. Enhanced green fluorescent protein (EGFP) dsRNA (dsEGFP) was used as the control for *RfARSB* dsRNA (dsARSB). For each larva, 1 µg of dsRNA was injected into the hemocoel. At 12 and 24 h post injection, salivary glands were collected to check the efficiency of RNAi by RT-qPCR, as described above. To explore the roles of *RfARSBs* in the biosynthesis of p-benzoquinone related to external immunity, based on the optimum principle of RNAi efficiency, we subsequently determined the antibacterial activity of OS against *E. coli* and *S. aureus* and the concentration of p-benzoquinone 12 h after injection of dsRNA.

### 4.8. Microbial Inhibition Assays

To test the strength of external immune efficiency in larvae, we detected the antimicrobial properties of larval OS through microbial inhibition assays against one fungus and two bacteria, including a gram-negative bacterium and a gram-positive bacterium. A disc diffusion method and a turbidimetric method were employed for the fungal and bacterial species, respectively.

More concretely, for the fungal inhibition assay, PDA plates (90 mm Ø) appropriate for *M. anisopliae* were prepared and inoculated with 2-mL conidial suspensions of cultured fungus (1.0 × 10^4^ conidia/mL). Sterile paper discs (6 mm Ø) containing 10-µL OS diluents, which were diluted one-fold with sterile water in advance, were placed onto the agar plates. The plates were kept for 72 h at 25 °C, and the diameter of each inhibition zone was recorded. However, for the antibacterial assay, we followed and modified the procedure described by Shi et al. (2014) [52]. Both *E. coli* and *S. aureus* were grown under the above conditions in LB medium. The experiments were performed in sterilized 96-well plates with a final volume of 130 μL. Then, 100-µL aliquots of bacterial culture were added to 30-µL OS samples serially diluted 20 times with sterile water. Subsequently, plates were incubated at 37 °C for 24 h. Bacterial growth was measured as the cell concentration, which was determined by measuring the OD_600_ using a SpectraMax 190 reader (Molecular Devices, Sunnyvale, CA, USA). In all microbial inhibition assays, the treatments with sterile water and tetracycline solution (10 mg/mL) served as the negative and positive controls, respectively.

### 4.9. Quantification of p-Benzoquinone in Larval Oral Secretions

Oral secretion samples were submitted to Shanghai Bioclouds Biological Technology Co., Ltd., Shanghai, China, for targeted metabonomic detection. To quantify the main external immune active component in the larval OS, 1,4-benzoquinone (p-benzoquinone, PBQ) (Sigma-Aldrich, St. Louis, MO, USA) was obtained from a commercial source. Then, authentic standard solutions (0.1 mg/mL p-benzoquinone diluted with methanol) were prepared, and a five-point calibration was performed by gas chromatography-mass spectrometry (GC-MS). On the basis of the standard curve, the areas of the abundances from GC-MS were transformed to masses. Samples were run on a 7890A gas chromatograph coupled with a 5975C mass spectrometer (Agilent, Palo Alto, CA, USA). Derivatized extracts (1 μL) were injected onto a nonpolar DB-5MS capillary column (30 m × 250 μm I.D., J&W Scientific, Folsom, CA, USA) using a G6500 CTC PAL autosampler (Agilent, Palo Alto, CA, USA), and the injection was run in pulsed splitless mode. The procedures and conditions for GC-MS were as described by Pu et al. (2020) in a previous study [32]. Each sample (100 mg) was composed of OS produced by 9 larvae, and six biological replications were carried out for each treatment.

### 4.10. Data Analysis

One-way analysis of variance (ANOVA) accompanied by Tukey’s honestly significant difference (HSD) multiple comparison test was employed to analyze the differences in the relative expression levels of *RfARSBs* across different tissues/organs and the antimicrobial activity of the larval OS. However, the differences in RNAi efficiency, quantity of p-benzoquinone in larval OS, and relative transcript levels of *RfARSBs* between larvae exposed to *M. anisopliae* and control larvae were examined by Student’s *t* test. The threshold for level of significance was set to 0.05 (*p* < 0.05). All data are expressed as the mean ± standard error (SE), and all statistical analyses were completed with IBM SPSS Statistics 21.0 software (SPSS Inc., Chicago, IL, USA).

## Supplementary Materials

Supplementary materials can be found at https://www.mdpi.com/1422-0067/21/5/1610/s1.
