# Supplementary material for: Identification of Novel ARSB Genes Necessary for p-Benzoquinone Biosynthesis in the Larval Oral Secretion Participating in External Immune Defense in the Red Palm Weevil
Source: Int J Mol Sci. 2020 Feb 26;21(5):1610. doi: 10.3390/ijms21051610 (PMC7084252; doi:10.3390/ijms21051610)
Supplement: Supplementary file 1 [file ijms-21-01610-s001.pdf]

```
1       AAGACATAGGCCCAGGGACGTTTGGGTTTTTGCATTGACCGTTCTCGATCTTCTTCCTCA
61      TCGACGGGGTGAAAGGGTTGGCGTGAAGATGAAGTGCTTAGTGTTGGTTGTTCTTGTGCT
-18                                  M  K  C  L  V  L  V  V  L  V  L
121     GGTGTTCGGTGGGGCGTTCACGGATAAGACCAAGAAGCCCCACATAGTTGTGATCATCGG
-7        V  F  G  G  A  F  T  D  K  T  K  K  P  H  I  V  V  I  I  G
181     AGATGATATGGGATTTAATGATGTCAGTTTTCATGGCAGCGATGAAATACCAACGCCAAA
14        D  D  M  G  F  N  D  V  S  F ‖H  G |S  D  E  I  P  T  P  N
241     CATTGACGCACTCGCATATAATGGAGTTATACTAAACAGTCATTACACGCAAGCTTTATG
34        I  D  A  L  A  Y  N  G  V  I  L  N  S  H  Y  T  Q  A  L  C
301     CACCCCTTCTAGAGCCGCTTTCCTCACTGGAAGATACCCTATACATCTTGGTATGCAACA
54        T  P  S  R  A  A  F  L  T  G  R  Y  P  I  H  L  G  M  Q  H
361     TCTAGTCATTTTGGAGCCTGAACCCTGGGGAGTACCACTTAACGAAACCCTTCTGCCCGA
74        L  V  I  L  E  P  E  P  W  G  V  P  L  N  E  T  L  L  P  E
421     ATACCTCAAGAGACATGGATATATCACCAGGGCTATCGGTAAATGGCACTTGGGATTTTG
94        Y  L  K  R  H  G  Y  I  T  R  A  I  G  K  W  H  L  G  F  C
481     TAAGAAGGAATACACGCCCACATATCGAGGTTTCGACAGTCATTATGGATACTGGCAAGG
114       K  K  E  Y  T  P  T  Y  R  G  F  D  S  H  Y  G  Y  W  Q  G
541     CTTCCATGACTACTACGAACACACTATTCATGCCACGTACACTTCGGAATTTGGATATGA
134       F  H  D  Y  Y  E  H  T  I  H  A  T  Y  T  S  E  F  G  Y  D
601     TATGCGTCGAAATATGAGCGTTGATTGGGAGGCCAAAGGAAAATATTCCACGGAGCTCTT
154       M  R  R  N  M  S  V  D  W  E  A  K  G  K  Y  S  T  E  L  F
661     CACTGACGAAGCGGTTAACACTATCCGGGACCACAACACCAAGGATCCCCTCTTTATGTA
174       T  D  E  A  V  N  T  I  R  D  H  N  T  K  D  P  L  F  M  Y
721     TTTGGCTCATCTGGCGCCTCACGCTGGCAACGACTGGGATCCTCTGCAGGCCCCTGATGA
194       L  A  H  L  A  P  H  A  G  N  D  W  D  P  L  Q  A  P  D  E
781     AGAAATAACCAAATTTGCTCATATCCAAGACCCAGAAAGAAGAATTTACGCCGCTATGGT
214       E  I  T  K  F  A  H  I  Q  D  P  E  R  R  I  Y  A  A  M  V
841     GTCAATGTTGGATAGAAGCGTGGGTGCTGTGGTGGGAGCCTTGAGGGAGAAGCACATGCT
234       S  M  L  D  R  S  V  G  A  V  V  G  A  L  R  E  K  H  M  L
901     GGAAAACTCCATTATCATCTTTATGTCAGACAACGGAGCTGCTCCAGAAGGTATCCACGC
254       E  N  S  I  I  I  F  M  S  D  N  G  A  A  P  E  G  I  H  A
961     CAACCACGGATCCAATTACCCATTTAGGGGGGCAAAGAATTCGGGCTGGGAAGGTGGTAT
274       N  H  G  S  N  Y  P  F  R  G  A  K  N  S  G  W  E  G  G  M
1021    GAGAAACATCGCTGCAATCTGGAGTCCTCTCATAAAGAAACCTCAAAGAGTCTCGAATAA
294       R  N  I  A |A  I  W  S  P  L  I  K  K  P  Q  R  V  S  N  N
1081    TCTGATGCACATCTCCGATTGGTTACCGACTTTGCTGTCCGCTGCAGGTTTGGGCACCTC
314       L  M  H  I  S  D  W  L  P  T  L  L  S  A  A  G  L  G  T  S
1141    CGAGTTACCATCATCCCTCGACGGTCGAGATCAGTGGAAGGCCATATCTGAGGGTTCCGA
334       E  L  P  S  S  L  D  G  R  D  Q  W  K  A  I ‖S  E  G  S  E
1201    ATCACCGAGAACTGAGATCTTGCACAACATCGACGATATCGACAAATACGGAGCCATACG
354       S  P  R  T  E  I  L  H  N  I  D  D  I  D  K  Y  G  A  I  R
1261    CCAAGGAGAATGGAAATATTTATACGGATCCGCGTCCAAAGGACGAATGGATTCGTGGTA
374       Q  G  E  W  K  Y  L  Y  G  S  A  S  K  G  R  M  D  S  W  Y
1321    CGGAAACAATGGAAAGGACCCTCTCTATAACTACGACGAAGGGATCGTGCTTCAATCACA
394       G  N  N  G  K  D  P  L  Y  N  Y  D  E  G  I  V  L  Q  S  Q
1381    AGCAGGAAGCGCCATATCCGGATTGATTACATTCCAACAGATCAAGGAAAAAAATTCGTT
414       A  G  S  A  I  S  G  L  I  T  F  Q  Q  I  K  E  K  N  S  L
1441    GCACGACCATCACCAAAAGATGAACTTCAGCGTCAACCTCCTGGACGCCGAAGACATCAT
434       H  D  H  H  Q  K  M  N  F  S  V  N  L  L  D  A  E  D  I  I
1501    ACGTTTAAGACAAGACGCTACTGTTAAGTGCAACGCGTCGAAAACCCAGAAAAAAGCAGA
454       R  L  R  Q  D  A  T  V  K  C  N  A  S  K  T  Q  K  K  A  E
1561    GTGCAAACCGATGGAGTCGCCCTGCTTGTTCAACATCAAGGACGACCCGTGCGAAACTGT
474       C  K  P  M  E  S  P  C  L  F  N  I  K  D  D  P  C  E  T  V
1621    CAATCTCATCGCTGAACATCCGAAGATAGCCGCCAGGTTGCAGGAAGAACTGTTAAATTA
494       N  L  I  A  E  H  P  K  I  A  A  R  L  Q  E  E  L  L  N  Y
1681    CAAAAAATCAACTCGCCCACCAATCAATATCCCCAGAGACCCCAAGGCCGATCCCTCCAG
514       K  K  S  T  R  P  P  I  N  I  P  R  D  P  K  A  D  P  S  R
1741    GTTCAACGGTACCTGGACCAACTGGCTAGATTATGATGTCACCCAAGAAAAGATCAGCTT
534       F  N  G  T  W  T  N  W  L  D  Y  D  V  T  Q  E  K  I  S  F
1801    CAACGCGCTCTCTCATTTGGCCGTTGGCTTGATTTCTGGAGCATGCGTAGCCGTGGTCGT
554       N  A  L  S  H  L  A  V  G  L  I  S  G  A  C  V  A  V  V  V
1861    CATAGTTCTGGTTCTTCTGACGATCACCTGTAGGAAAACGCCAAAACGGAGCATGCTGGG
574       I  V  L  V  L  L  T  I  T  C  R  K  T  P  K  R  S  M  L  G
1921    ACTGTACGAAGAGGCGGAAATGTGTACGGAGTTGGAGACCAAATCGGACAATACCTTTGA
594       L  Y  E  E  A  E  M  C  T  E  L  E  T  K  S  D  N  T  F  E
1981    GGAGAGAGAAAGACAGATGAGAGCGTCGCTCAGGAGCGAGATCAGAGACGTATGATTTGT
614       E  R  E  R  Q  M  R  A  S  L  R  S  E  I  R  D  V  *
2041    GTTCAGTGATTGTGTCGCTGCCTCTGTGTTCTTTTATAGGAAACTTAGTTGTATAAGTTA
2101    ATTTATTATAAA
```

Figure S1. Nucleotide and amino acid sequences of RfARSB-0311. The predicted signal peptide is underlined in black. The regions between the two "‖" and two "|" indicate the core functional domains (Pfam), namely, sulfatase and phosphodiesterase, respectively. The transmembrane region is highlighted with a red underline. Putative N-linked and O-linked glycosylation sites are labeled with "●" and "●", respectively. The most highly conserved cysteine residues are circled in red.

```
1       TACCATATTTCTTGTTTTGTTTTAAAAGCTTAGTGAATAATAGTGTAAAGTGATATATGA
61      TGGAAAGATGGCTAGTGCTAATTGGATTTGTTATTTTACATACGTCCAGGTGTGGGGTAT
-18     M  E  R  W  L  V  L  I  G  F  V  I  L  H  T  S  R  C  G  V
121     ATTCAGCTGAACAGCCTAATATTATATTTATTATTGTGGATGATTTGGGCTGGAACGATG
3       Y  S  A  E  Q  P  N  I  I  F  I  I  V  D  D  L  G  W  N  D
181     TGGGATTTCATGGAAGCAACCAGATACCTACGCCAAATATTGACGTTTTGGCTTACAATG
23      V  G  F ||H  G |S  N  Q  I  P  T  P  N  I  D  V  L  A  Y  N
241     GCATTATACTCAACAGTCACTATGTGCAGTCTTATAGCACACCAACGAGGGCGGCCCTTC
43      G  I  I  L  N  S  H  Y  V  Q  S  Y  S  T  P  T  R  A  A  L
301     TCTCGGGAATATATCCAATGAAGTTAGGTATGCAAGGGCCAAGTTTTTTGGCAGCCGAGA
63      L  S  G  I  Y  P  M  K  L  G  M  Q  G  P  S  F  L  A  A  E
361     AGAAAGCTATGCCAAAACATAAACTTTTACCAGAATATTTTAAGGATATGGGCTACGAGA
83      K  K  A  M  P  K  H  K  L  L  P  E  Y  F  K  D  M  G  Y  E
421     CTCATCTTGTTGGCAAATGGCATTTGGGATACAGTAGATGGAACGAGACACCGACCTTGC
103     T  H  L  V  G  K  W  H  L  G  Y  S  R  W  N  E  T  P  T  L
481     GAGGTTTTGATCACCATTTCGGATTTTACAACTCGTATTGCAGTTATTATGATTATTTAT
123     R  G  F  D  H  H  F  G  F  Y  N  S  Y  C  S  Y  Y  D  Y  L
541     CAACTTGGACTTACAATGAACACGATTATACTGGTTTCGAACTCAGGAAAGATGGTCAAG
143     S  T  W  T  Y  N  E  H  D  Y  T  G  F  E  L  R  K  D  G  Q
601     CAGTGTTTGATGAGGCTGGAAAATATGCGACAGATCTTTTCACTGAATACACAGTCAATA
163     A  V  F  D  E  A  G  K  Y  A  T  D  L  F  T  E  Y  T  V  N
661     CTATCAATGAGCATGATAGTGGGAAACCTTTATTCCTCATGTTATCACATTTAGGTGTTC
183     T  I  N  E  H  D  S  G  K  P  L  F  L  M  L  S  H  L  G  V
721     ATGCAGCCAATAGCGGCAAACCTTTGGAGGCACCACAAGAAACTATAAATACGTTTCGAC
203     H  A  A  N  S  G  K  P  L  E  A  P  Q  E  T  I  N  T  F  R
781     ATATAGTTGATGCTAATAGAAGAACATACGCAGCAATGGTATCCAAGATAGATGATAGTA
223     H  I  V  D  A  N  R  R  T  Y  A  A  M  V  S  K  I  D  D  S
841     TGGGTTCAATCATTACCGCTTTAGATCAGAAGAATATGTTATCAAATACTATTATAGCTT
243     M  G  S  I  I  T  A  L  D  Q  K  N  M  L  S  N  T  I  I  A
901     TTATCAGTGACAATGGTGCTCCTACTACTGGTCCCTACCAAAATTGGGGTAGTAATTTAC
263     F  I  S  D  N  G  A  P  T  T  G  P  Y  Q  N  W  G  S  N  L
961     CTTTGAGAGGGATTAAAGATACACTTTTTGAAGGTGGTGTTAGGTCCGTCGCATTCATCT
283     P  L  R  G  I  K  D  T  L  F  E  G  G  V  R  S  V  A  F  I
1021    GGAGCCCTTTGCTTGTACAAACTGCACGAGTATCTACAGATCTAATGCATGTTACCGACT
303     W  S  P  L  L  V  Q  T  A  R  V  S  T  D  L  M  H  V  T  D
1081    GGTTCCCAACTTTATTTACGGCTGCTGGTGGTGACATCGATGCACTAGATCCTGAATTGG
323     W  F  P  T  L  F  T  A  A  G  G  D |I  D  A  L  D  P  E  L
1141    ATGGTATCGATGTTTGGTCTAGTTTGGTTTATGATCTACCATCACCAAGAAATGATATTT
343     D  G  I  D  V  W  S  S  L ||V  Y  D  L  P  S  P  R  N  D  I
1201    TGCTTAATATTGACGAAAAAACAAGAAATGCCGGATTAAGGTTTTATAATTGGAAACTCA
363     L  L  N  I  D  E  K  T  R  N  A  G  L  R  F  Y  N  W  K  L
1261    TTGTAGGCACTAGTCAAAATGGAAGTTATAATGATTATTTTGGGCATATACCCATGGAGA
383     I  V  G  T  S  Q  N  G  S  Y  N  D  Y  F  G  H  I  P  M  E
1321    ATATCGAAGAGATACCATACAATTCATCAGCAGTTTATGAAAGTCCAGCTGGACAAGTGT
403     N  I  E  E  I  P  Y  N  S  S  A  V  Y  E  S  P  A  G  Q  V
1381    TAAAGAAAGTTAATTACAGTCCATTAAGTGAAGTTGAATATGAAAATATAAGATATCAAG
423     L  K  K  V  N  Y  S  P  L  S  E  V  E  Y  E  N  I  R  Y  Q
1441    CAACAATAAAATGTACAGCCACTAAGAAGAATCCATGTGATCCAGCAACCGGTGCAGTTT
443     A  T  I  K  C  T  A  T  K  K  N  P  C  D  P  A  T  G  A  V
1501    GTTTGTATGATATACCGAGCGATCCTTGCGAAGAAAATGATCTTGCAAAATACTTTCCAA
463     C  L  Y  D  I  P  S  D  P  C  E  E  N  D  L  A  K  Y  F  P
1561    GCGTCGTTAGGAGAATGAAACGGGCTTTAGTCGATTATAGAAAAGGCCTCATACCTCAAA
483     S  V  V  R  R  M  K  R  A  L  V  D  Y  R  K  G  L  I  P  Q
1621    TAGAGGGTGAGGTTGATATTGAAGCTGCGGATCCTAAGCTGTTTAAATATACTTGGTCGC
503     I  E  G  E  V  D  I  E  A  A  D  P  K  L  F  K  Y  T  W  S
1681    CATGGGTAGACTGTGCCGACGCCACCTGTAATATTGCTTAGTATGTGCCTTGTAATTTTG
523     P  W  V  D  C  A  D  A  T  C  N  I  A  *
1741    TTGCATATTTGATAGTTTTAATCTATAAGTTATAAATAAACTATAGTTAGAATTAGTTAA
1801    CATATGATTTACCC
```

Figure S2. Nucleotide and amino acid sequences of RfARSB-11581. The predicted signal peptide is underlined in black. The regions between the two "‖" and two "|" indicate the core functional domains (Pfam), namely, sulfatase and phosphodiesterase, respectively. Putative N-linked and O-linked glycosylation sites are labeled with "●" and "●", respectively. The most highly conserved cysteine residues are circled in red.

```
1      TCTCAAGCCGGCGTCGAAATCGCACTTAAACGCTTTTAAATTTCGGTTATTACCAGCCCA
61     CACTGACGTTCTACATGCATCCGAGTACAAAAATAAACAAATTGAACGAGACTTGAAACC
121    GAGATGGCCGGTATTCCGTGTGGTGTGCGGGCATTGCTTTTGATATTGTTCTTTGTGTGC
-21      M  A  G  I  P  C  G  V  R  A  L  L  L  I  L  F  F  V  C
181    GATTCCAGCCAGTACAAATCGCCGCACATCATTTTTATCGTCGCCGATGATTTAGGATTT
-2      D  S  S  Q  Y  K  S  P  H  I  I  F  I  V  A  D  D  L  G  F
241    AATGACGTAGGTTTCCATGGATCCGGACAAATTCCCACACCCAATATTGATGCCCTAGCA
19      N  D  V  G  F  H  G  S||G  Q  I  P  T  P  N  I  D  A  L  A
301    TACTCGGGATTGATCTTAAACAAGTACTACGTGAATCCAATATGTACCCCATCCAGGAGC
39      Y  S  G  L  I  L  N  K  Y  Y  V  N  P  I  C  T  P  S  R  S
361    GCCTTGATGACGGGCAAATACCCTATCCGCACGGGCATGCAACACACCGTGCTCTTCGGA
59      A  L  M  T  G  K  Y  P  I  R  T  G  M  Q  H  T  V  L  F  G
421    GCGGAACCGAGGGGTTTGCCTTTGTCTGAAAAAATCCTGCCCCAGTATCTAAGGGAGTTA
79      A  E  P  R  G  L  P  L  S  E  K  I  L  P  Q  Y  L  R  E  L
481    GGGTACGTGAACAGGATAGTCGGTAAATGGCACCTAGGCTCTTGGAGGAAGGAGTACACG
99      G  Y  V  N  R  I  V  G  K  W  H  L  G  S  W  R  K  E  Y  T
541    CCCTTGTACCGGGGTTTCCAGTCTCATCTTGGTTACTGGACCGGTCACCAGGACTACTTT
119     P  L  Y  R  G  F  Q  S  H  L  G  Y  W  T  G  H  Q  D  Y  F
601    GACCACATCGCAATGGAGAACGGCCAGTGGGGCTTGGATATGAGACGCAACCTGTCCGTC
139     D  H  I  A  M  E  N  G  Q  W  G  L  D  M  R  R  N  L  S  V
661    GCTTACGATCTTCATGGTAAATACTCGACTGACATCTTCACAGAGGAAGCAGTGAAGATA
159     A  Y  D  L  H  G  K  Y  S  T  D  I  F  T  E  E  A  V  K  I
721    ATCAAAGAACACAACCAAGAACATCCCCTGTTTTTATACATGGCTCATGCAGCGGTACAT
179     I  K  E  H  N  Q  E  H  P  L  F  L  Y  M  A  H  A  A  V  H
781    TCTGGCAACCCTTACAACCCTCTACCCGTATTGGACGCTGACGTCATGAAAATCAATGAT
199     S  G  N  P  Y  N  P  L  P  V  L  D  A  D  V  M  K  I  N  D
841    ATCGACGACTACAACCGGAGGCGCTTTGCAGCCATGATGAGCAAATTGGATCAGTCTGTC
219     I  D  D  Y  N  R  R  R  F  A  A  M  M  S  K  L  D  Q  S  V
901    GGGGAAGTGGTGAAAGCCCTCCAGGATCAAGACATGCTGAAGAACAGTATTATTGTCTTC
239     G  E  V  V  K  A  L  Q  D  Q  D  M  L  K  N  S  I  I  V  F
961    ACCACTGACAACGGAGGACCTGCAGCCGGTTTCAACCTAAACGCCGCCTCCAACTACCCC
259     T  T  D  N  G  G  P  A  A  G  F  N  L  N  A  A  S  N  Y  P
1021   CTGAGAGGGGTGAAAAATACTCTCTTCGAAGGTGGCGTCAGAGGGGCAGGACTAGTTTGG
279     L  R  G  V  K  N  T  L  F  E  G  G  V  R  G  A  G  L  V  W
1081   TCTCCACTAATAAAAAAACCGTCGAGAGTTTCTAATCAGTTCATGCACATAGTTGACTGG
299     S  P  L  I  K  K  P  S  R  V  S  N  Q  F  M  H  I  V  D  W
1141   TTACCTACCCTACTGGAAGCAGCTACAGGAGATATAACCAATATAACGAACTTGGATGGA
319     L  P  T  L  L  E  A  A  T  G  D  I  T  N  I  T  N  L  D  G
1201   GTGAGTATATGGAAATCTCTATCTCTAGACCAATCATCTCCCCGAACAGAAGTTTTACAC
339     V  S  I  W  K  S  L  S  L  D  Q||S  S  P  R  T  E  V  L  H
1261   AATATTGATGACATTTACGGAAATGCTGCGATAACTATCGATGAGTGGAAACTAGTGCAA
359     N  I  D  D  I  Y  G  N  A  A  I  T  I  D  E  W  K  L  V  Q
1321   GGTTCCACTTATAGTGGACAATGGGACTTTTGGTATGGTCCTGACGGACGAAACTACCCC
379     G  S  T  Y  S  G  Q  W  D  F  W  Y  G  P  D  G  R  N  Y  P
1381   TACAATATAACCCTAGTACAAGAGAGTCTATCAGGTAAGGCTCTGGAAAGTATCAATCGA
399     Y  N  I  T  L  V  Q  E  S  L  S  G  K  A  L  E  S  I  N  R
1441   GGTACCCCGGAAAGTACCATAACCAAACTAAGAGAAGAAGCCACTGTGATATGTAATAAT
419     G  T  P  E  S  T  I  T  K  L  R  E  E  A  T  V  I  C  N  N
1501   AGTCGGATAATATCTTGTAATGCTATCGAGAAGCCGTGTCTGTTCGACATCATCAGCGAC
439     S  R  I  I  S  C  N  A  I  E  K  P  C  L  F  D  I  I  S  D
1561   CCCTGCGAATACCAAAATTTTGCGGAGAAGTATCCACATATCGTAGAAAAACTACAAACC
459     P  C  E  Y  Q  N  F  A  E  K  Y  P  H  I  V  E  K  L  Q  T
1621   AGATTAAAGGAATACAATGCCACTGCAATACCACCAGGAAATCTGCCATTGGACGAAAGA
479     R  L  K  E  Y  N  A  T  A  I  P  P  G  N  L  P  L  D  E  R
1681   GGAAACCCAAAGTATTGGAACTATGTCTTTACGAATTTTGGAGACTATGACACAAACTCT
499     G  N  P  K  Y  W  N  Y  V  F  T  N  F  G  D  Y  D  T  N  S
1741   ATAATATTGATTTAGGTG
519     I  I  L  I  *
```

Figure S3. Nucleotide and amino acid sequences of RfARSB-14322. The predicted signal peptide is underlined in black. The region between the two "‖" indicates the core functional domain (Pfam), namely, sulfatase. Putative N-linked and O-linked glycosylation sites are labeled with "●" and "●", respectively. The most highly conserved cysteine residues are circled in red.

Table S1. Primers used in this study.

Name	Sequence (5′–3′)
**Primers for normal PCR**	
*Rf*ARSB-0311-F	ACTCCCCAGGGTTCAGGCTCCAA
*Rf*ARSB-0311-R	GACCCGTGCGAAACCGTCAATCT
*Rf*ARSB-11581-F	TCCGTCGCATTCATCTGGAGCCC
*Rf*ARSB-11581-R	ACAGGTGGCGTCGGCACAGTCTA
*Rf*ARSB-14322-F	TTCAACCTAAACGCCGCCTCC
*Rf*ARSB-14322-R	ACGGACAGGTTGCGTCTCATA
**Primers for RACE PCR**	
*Rf*ARSB-0311-3′ outer	GATTACGCCAAGCTTTCGCTGAACATCCGAAGATAGCCGCCA
*Rf*ARSB-0311-3′ inner	GCCTAGCCGTGGTCGTCATAGTTCTG
*Rf*ARSB-11581-3′ outer	TTAGGAGAATGAAACGGGCTTTAGTC
*Rf*ARSB-11581-3′ inner	GGTAGACTGTGCCGACGCCACCTGTA
**Primers for complete length**	
*Rf*ARSB-0311 QC-F	TAGTGAATAATAGTGTAAAGTGA
*Rf*ARSB-0311 QC-R	AGCAGTGGTATTAACGCAGAGTA
*Rf*ARSB-11581 QC-F	GGTTTTTGCATTGACCGTTCT
*Rf*ARSB-11581 QC-R	TAGGGCAAGCAGTGGTATCAA
**Primers for RT-qPCR**	
*Rf*ARSB-0311-RT-5′	GATAGAAGCGTGGGTGCTGT
*Rf*ARSB-0311-RT-3′	TGGTTGGCGTGGATACCTTC
*Rf*ARSB-11581-RT-5′	ACAGTAGATGGAACGAGACACCG
*Rf*ARSB-11581-RT-3′	CCAGCCTCATCAAACACTGCTTG
*Rf*ARSB-14322-RT-5′	CATTCTGGCAACCCTTACAACCC
*Rf*ARSB-14322-RT-3′	CTTCCCCGACAGACTGATCCAAT
GAPDH-5′	CCAAGGGAGCCAAGCAATT
GAPDH-3′	CGCTGATGCCCCTATGTATGT
**Primers for dsRNA synthesis**	
*Rf*ARSB-0311-ds-F	TAATACGACTCACTATAGGGGGATAAGACCAAGAAGCCCC
*Rf*ARSB-0311-ds-R	TAATACGACTCACTATAGGGCGCTCATATTTCGACGCATA
*Rf*ARSB-11581-ds-F	TAATACGACTCACTATAGGGCAAGAAATGCCGGATTAAGG
*Rf*ARSB-11581-ds-R	TAATACGACTCACTATAGGGTACCCATGGCGACCAAGTAT
*Rf*ARSB-14322-ds-F	TAATACGACTCACTATAGGGAGGGGCAGGACTAGTTTGGT
*Rf*ARSB-14322-ds-R	TAATACGACTCACTATAGGGACACGGCTTCTCGATAGCAT
